# Editorial Perspective: Rapid responses to understand and address children and young people’s mental health in the context of COVID‐19

**DOI:** 10.1111/jcpp.13626

**Published:** 2022-05-04

**Authors:** Cathy Creswell

**Affiliations:** ^1^ 6396 Departments of Psychiatry and Experimental Psychology University of Oxford Oxford UK

## Abstract

Prior to the pandemic, we already had good reason to be concerned about the mental health of children and young people. As an example, the 2017 Mental Health of Children and Young People (MHCYP) survey in England, comprising a large, national probability sample, identified that one in nine children had a probable mental health disorder, with a 49% increase in emotional disorders compared to a previous survey in 2004 (Sadler et al., 2018). The pandemic has clearly brought a broad range of challenges to children and young people. These include the direct viral threat to self, friends, and family (with recent estimates of a 17.5%–20.2% increase in parental bereavement in the United States; Kidman et al, 2021), as well as disruptions to school work, social interactions, family pressures, economic impacts, a lack of opportunity and ongoing uncertainty, and reduced access to mental health and other support from outside the home. So how have these experiences affected the mental health of children and young people?

Prior to the pandemic, we already had good reason to be concerned about the mental health of children and young people. As an example, the 2017 Mental Health of Children and Young People (MHCYP) survey in England, comprising a large, national probability sample, identified that one in nine children had a probable mental health disorder, with a 49% increase in emotional disorders compared to a previous survey in 2004 (Sadler et al., 2018). The pandemic has clearly brought a broad range of challenges to children and young people. These include the direct viral threat to self, friends, and family (with recent estimates of a 17.5%–20.2% increase in parental bereavement in the United States; Kidman, Margolis, Smith‐Greenaway, & Verdery, 2021), as well as disruptions to school work, social interactions, family pressures, economic impacts, a lack of opportunity and ongoing uncertainty, and reduced access to mental health and other support from outside the home. So how have these experiences affected the mental health of children and young people?

Unfortunately, we can never answer this question with complete certainty. The best data that we have on the prevalence of mental health problems during the pandemic in England comes from a repeat of the MHCYP conducted in July 2020, when England was under moderate restrictions to curb the further spread of COVID‐19 (NHS Digital, 2020). This survey indicated that the rate of mental health problems in children and young people had increased to one in six. Of note, a similar increase in probable mental health problems was found for primary and secondary school‐aged participants, with a particular increase in difficulties for primary school‐aged boys (and particularly for hyperactivity/inattention problems). Given findings that more young people with mental health difficulties reported that lockdown restrictions had made their life worse, than better, it seems likely that the pandemic had contributed to this increase. However, the context of already increasing levels of mental health problems and the absence of data immediately prior to the pandemic, with which to make comparisons, limits firm conclusions. As such, it is critical that we triangulate these findings with those from studies with other designs and methods that are not able to tell us about *prevalence* but can help us to understand patterns of change over time, and how those may have differed for children and young people with different characteristics and living in different circumstances.

The importance of being able to refer to prepandemic data has been highlighted in some calls to action (e.g. Pierce et al., 2020), and helpfully, some research teams were quick to respond by following up on existing cohorts to establish how mental health symptoms had changed over time. To date, however, these studies have provided mixed results. For example, in the United Kingdom one study reported a significant reduction in anxiety symptoms, an increase in well‐being, and no change in depression among 13–14‐year‐olds assessed during the first full national lockdown (April/May 2020) in comparison to data collected in October 2019 (Widnall et al., 2020), while another reported an increase in symptoms of depression and no significant change in anxiety among 7–11‐year‐olds tested during the first full lockdown, in comparison to data collected between June 2018 and September 2019 (Bignardi et al., 2020). These mixed findings are likely influenced by the nature of these particular samples and the timing of assessments, but the interpretation is also complicated by the fact that how children and young people respond to mental health measures varies considerably and quickly with age. A clear example comes from the widely used Revised Child Anxiety and Depression scale where there is more than a four‐point difference in mean scores for children in grades 3 and 4 compared to those in grades 5 and 6 (based on US samples; Chorpita, Moffitt, & Gray, 2005).

In an attempt to shed more light on the questions raised about the timing of assessments and the variation in children and young people’s experiences, we have been running the longitudinal COVID‐19: Supporting Parents, Adolescents, and Children during Epidemics (Co‐SPACE) study at monthly intervals since the first week of the UK lockdown in March 2020 (Waite, Creswell, & Praveetha, 2020). To date over 8000 parents of 4–16‐year‐olds from across the United Kingdom have provided sufficient data for us to assess child mental health outcomes, with self‐report data from a subset of adolescents. We are always at great pains to emphasise that this is not a nationally representative sample – it was a convenience sample recruited through social media and extensive support from our UKRI Emerging Minds Research network partners. Indeed, the sample is notably more affluent than the general population, with only 9% of the population living below the poverty line compared to 19% of the national population, and it is critical that this is held in mind when interpreting the data. Nevertheless, what we have seen among this group of participants has followed clear patterns. First, and consistent with MHCYP findings, low family income and the presence of special educational needs and/or neurodevelopmental disorders among children have consistently been associated with elevated child mental health symptoms within our Co‐SPACE population throughout the pandemic. Second, mental health symptoms among primary school‐age children (and the proportion for whom these are causing interference in day to day life) have increased at times of maximum restrictions (when most children have been learning from home and have been unable to mix with children from other households) and this has been particularly pronounced for hyperactivity/inattention symptoms (also in line with the MHCYP data, and potentially suggesting that those findings may have underrepresented the extent of problems when restrictions were at their highest). The pattern among secondary school‐aged participants have been more stable among the Co‐SPACE population, but with concerning increases in difficulties when we entered a second national lockdown in January/February 2021 (Creswell et al., 2021).

It is important to note that, to date, our findings have mostly been based on parent/carer reports, however, we have been reassured to see that our adolescent self‐report data have been broadly consistent (e.g. Pearcey, Shum, Waite, Patalay, & Creswell, 2020). While the more dramatic initial changes among younger participants may appear surprising, given we know that adolescence is a time of particular vulnerability for the emergence of mental health problems (e.g. Blakemore, 2019), they potentially reflect difficulties for younger children maintaining friendships remotely, alongside increases in family stress as a result of home‐schooling. Eighty‐five per cent of the secondary school age participants in the Co‐SPACE study regularly communicated with peers throughout the first lockdown, compared to only 33% of the primary school age group (Pearcey, Raw, Shum, Waite, & Creswell, 2020). Amongst this study sample, far more parents of younger than older children reported not being able to meet the needs of both their work and their child when most children were working from home, and parents self‐reported stress was substantially higher (Shum, Skripkauskaite, Pearcey, Waite, & Creswell, 2021). Ongoing work will dig into the extent to which variation in these sorts of social and family experiences may account for differences in the trajectories of change in children’s mental health symptoms over time, and accompanying qualitative work is proving invaluable in getting a deeper understanding of the patterns that we have seen.

While we continue to learn about how things have changed for whom, what is clear from the different sources of data that we now have is that, although many children and young people have done well throughout the pandemic, times of heightened restrictions have been associated with an increase in the numbers who have struggled with particularly high rates among children and young people who had preexisting vulnerabilities (Waite et al., 2021). Unfortunately, this has been accompanied by new challenges in accessing mental health support due to pandemic‐related restrictions, in a context in which access was already extremely patchy, and often poor, across the country (Children’s Commissioner, 2020). While there is still a huge amount to do to address the barriers families face in accessing evidence‐based support, I was pleased to be able to end my talk with a few potential solutions. These include the UKRI funded SPARKLE trial which is embedded within the Co‐SPACE study and is evaluating a public health app to support parents and carers with day to day parenting challenges (Kostyryka‐Allchorne et al., 2021); the MRC/NIHR funded Co‐CAT trial which is comparing a therapist guided, brief online‐programme for child anxiety problems (based on a guided parent‐led approach) to treatment as usual in child and adolescent mental health services in the pandemic context (ISRCTN 12890382); and the MRC funded CoRAY project which involves working with adolescents to create resources to address their priorities for mental health support (Emerging Minds, 2020). Evaluations are ongoing but we hope that ultimately these evidence‐informed, efficient, accessible, and codesigned solutions may go some way to addressing some of the needs and barriers faced by families during the pandemic and beyond.
